# Genome Sequences of Marichromatium gracile HOL-1 and Its Purple Photosynthetic Coisolate, *Afifella* sp. H1R

**DOI:** 10.1128/mra.00033-22

**Published:** 2022-03-08

**Authors:** J. A. Kyndt, S. Dubey, N. Frazier, T. E. Meyer

**Affiliations:** a College of Science and Technology, Bellevue University, Bellevue, Nebraska, USA; b Department of Chemistry and Biochemistry, the University of Arizona, Tucson, Arizona, USA; University of Southern California

## Abstract

We sequenced the genomes of both the purple sulfur gammaproteobacterium Marichromatium gracile HOL-1 and another purple photosynthetic organism, strain H1R, that was originally isolated as an unidentified contaminant. Through genome sequencing, we have now identified organism H1R as a species of *Afifella*. A whole-genome-based phylogenetic analysis of both species is provided.

## ANNOUNCEMENT

Strain H1R was isolated as a contaminant of Marichromatium gracile HOL-1, and although it was known to be a photosynthetic purple nonsulfur bacterium, its true nature has been elusive for decades. The species was simply referred to as “organism H1R” (red isolate from HOL-1), a purple photosynthetic bacterium of unknown taxonomy (1–3). It was found to contain two soluble cytochromes (*c*_5_ and *c*_3_); however, the closest homologue of the *c*_5_ cytochrome appeared to be from *Shewanella* (2), which is not a purple photosynthetic bacterium, indicating that there may have been another contaminant. We have now sequenced the genomes of both Marichromatium gracile HOL-1 and the isolated H1R species.

Marichromatium gracile HOL-1 was originally isolated in 1980 from marine waters from the Southern California coast and was cultivated on Pfennig’s medium I (DSMZ medium 28), in an effort to study novel cytochrome *c* proteins and photosynthetic mechanisms of purple sulfur bacteria. Strain H1R strain had been separated from it by one of us (T.E.M.) decades ago, by repetitive plating on *Rhodospirillaceae* medium (DSMZ medium 27) supplemented with 3% NaCl. This separation occurred before strain HOL-1 was deposited as a pure culture into the DSMZ culture collection (DSM 1712). Genomic DNA was isolated from glycerol freezer stocks of both species. DNA was purified using the GeneJET DNA purification kit (Thermo Scientific). Qubit and NanoDrop DNA analyses showed 260/280 nm absorbance ratios of 1.81 for strain HOL-1 and 1.60 for strain H1R. The sequencing libraries were prepared using the Illumina Nextera DNA Flex library prep kit. The genomes were sequenced with an Illumina MiniSeq using 500 μL of a 1.8 pM library. Paired-end (2 × 150 bp) sequencing was performed, and the number of obtained reads is given in Table 1. Quality control of the reads was performed using FASTQC within Basespace (Illumina; version 1.0.0), using a k-mer size of 5 and contamination filtering. We assembled the genomes *de novo* using Unicycler (version 0.4.8) through PATRIC (4, 5). The results of this assembly approach are summarized in Table 1. The HOL-1 genome was 3,749,065 bp in length, with a GC content of 68.49%, while the H1R genome was 4,233,774 bp in length, with a GC content of 63.72%. The genomes were annotated using the NCBI Prokaryotic Genome Annotation Pipeline (PGAP) (6–8). An EvalG genome quality analysis, using the checkM algorithm (9), showed an estimated 100% completeness and 0% contamination for both genomes (Table 1). Default parameters were used for all software unless otherwise noted.

**TABLE 1:** Overview of the properties of the Marichromatium gracile HOL-1 and *Afifella* sp. H1R whole-genome sequences

Organism	No. of reads	Genome length (bp)	No. of contigs	*N*_50_ (bp)	GC content (%)	No. of genes	No. of tRNAs	Coverage (×)	Completeness (%)	GenBank accession no.
Marichromatium gracile HOL-1	1,872,258	3,749,065	68	144,724	68.49	3,330	48	75	100	JAKEDQ010000000
*Afifella* sp. H1R	2,828,530	4,233,774	10	2,471,581	63.72	3,973	50	101	100	JAKEDY010000000

**FIG 1:**
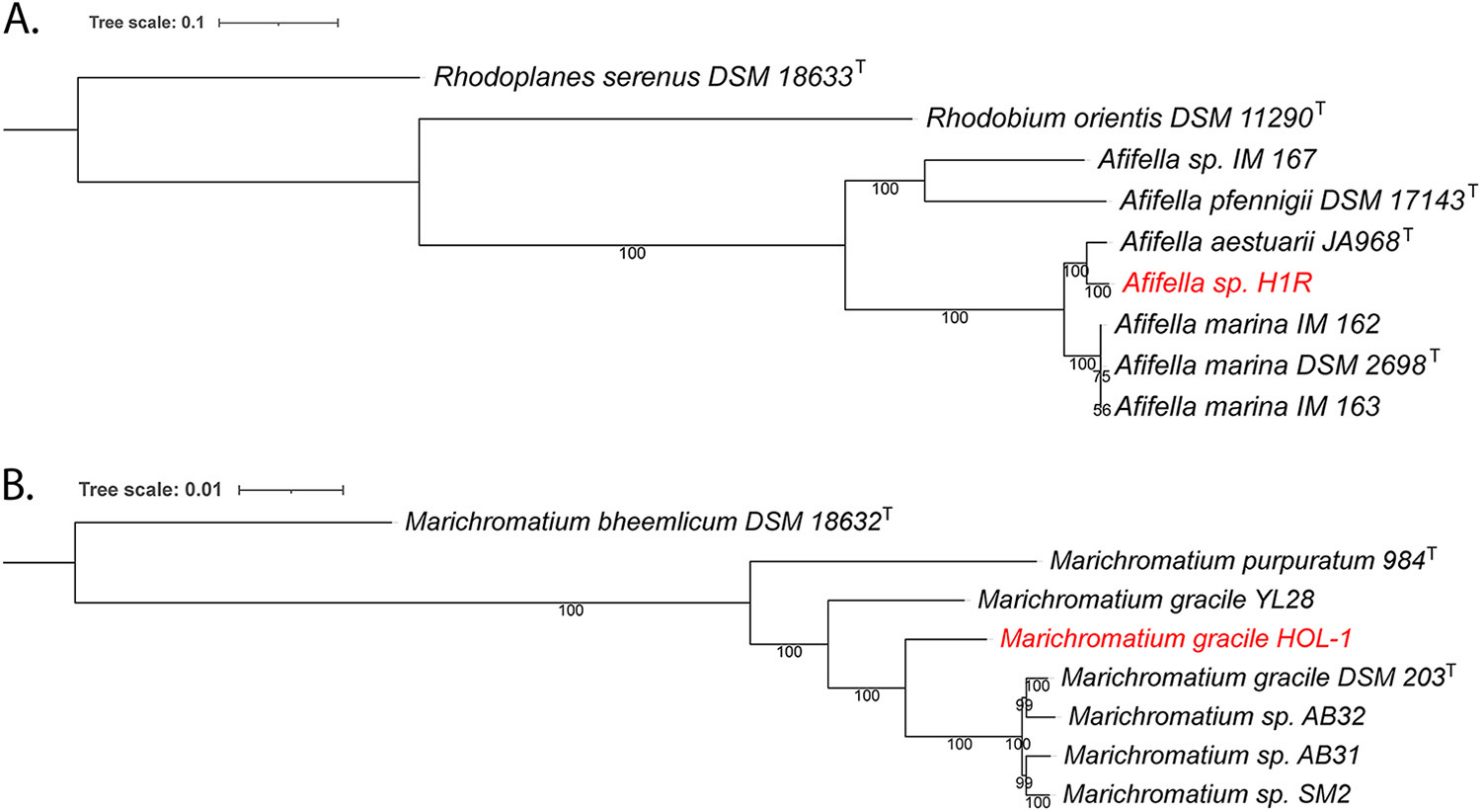
(A) Whole-genome-based phylogenetic tree of *Afifella* sp. H1R and its closest relatives; (B) whole-genome-based phylogenetic tree of *Marichromatium* related species. The phylogenetic trees were generated using the CodonTree method within PATRIC (5), which used PGFams as homology groups. Five hundred (A) and 1,000 (B) PGFams were used from these selected genomes using the CodonTree analysis, and the aligned proteins and coding DNA from single-copy genes were used for RAxML analysis (10, 11). The support values for the phylogenetic tree were generated using 100 rounds of the rapid-bootstrapping option of RAxML (5). Rhodoplanes serenus DSM 18633^T^ and Marichromatium bheemlicum DSM 18632^T^ were used as an outgroups. Interactive Tree Of Life (iTOL) was used for the tree visualization (15).

Both a whole-genome phylogenetic analysis using RAxML (10, 11) (Fig. 1A) and a JSpecies comparison (12) of average nucleotide identity (ANI) showed that H1R belongs to the genus *Afifella* (formerly *Rhodobium*). The closest relative, with an ANI of 95.5%, is Afifella aestuarii JA968 (13). This is close to the arbitrary species cutoff of 95% (12), and further physiological studies will be needed to determine a possible species differentiation. The Marichromatium gracile HOL-1 whole-genome comparison (Fig. 1B) showed Marichromatium gracile DSM 203^T^ (14) as its closest relative, with an ANI of 97.4%, confirming its species placement. Besides expanding the relative small number of genomes available for these genera (6 and 7 in NCBI GenBank), these new genome sequences and the whole-genome-derived phylogenetic placement help elucidate the previous cytochrome *c* analysis of Marichromatium gracile HOL-1 and its, up until now, unidentified coisolate (1–3).

### Data availability.

This whole-genome shotgun project has been deposited at DDBJ/ENA/GenBank under the accession numbers JAKEDQ000000000 (Marichromatium gracile HOL-1) and JAKEDY000000000 (*Afifella* sp. H1R). The versions described in this paper are versions JAKEDQ010000000 (HOL-1) and JAKEDY010000000 (H1R). The raw sequencing reads have been submitted to SRA, and the corresponding accession numbers are SRR17553093 (HOL-1) and SRR17553527 (H1R).
